# Extracellular high molecular weight α-synuclein oligomers induce cell death by disrupting the plasma membrane

**DOI:** 10.1038/s41531-023-00583-0

**Published:** 2023-09-28

**Authors:** Naohito Ito, Mayumi Tsuji, Naoki Adachi, Shiro Nakamura, Avijite Kumer Sarkar, Kensuke Ikenaka, César Aguirre, Atsushi Michael Kimura, Yuji Kiuchi, Hideki Mochizuki, David B. Teplow, Kenjiro Ono

**Affiliations:** 1 Department of Pharmacology, School of Medicine, Showa University, Tokyo, 142-8555 Japan; 2 Department of Internal Medicine, Division of Neurology, School of Medicine, Showa University, Tokyo, 142-8666 Japan; 3 Pharmacological Research Center, Showa University, Tokyo, 142-8555 Japan; 4 Department of Physiology, School of Medicine, Showa University, Tokyo, 142-8555 Japan; 5 Department of Oral Physiology, School of Dentistry, Showa University, Tokyo, 142-8555 Japan; 6 Department of Developmental Biology, Cincinnati Children’s Hospital Medical Center, Cincinnati, OH 45229-3026 USA; 7 Department of Neurology, Graduate School of Medicine, Osaka University, Osaka, 565-0871 Japan; 8 Brain Research Institute Center for Integrated Human Brain Science, Department of Functional Neurology and Neurosurgery, Niigata University, Niigata, 951-8122 Japan; 9 Department of Neurology, David Geffen School of Medicine, University of California-Los Angeles (UCLA), Los Angeles, LA 10833 USA; 10 Department of Neurology, Kanazawa University Graduate School of Medical Sciences, Kanazawa University, Kanazawa, 920-8640 Japan

**Keywords:** Cell biology, Proteins

## Abstract

α-Synuclein (αS), the causative protein of Parkinson’s disease and other α-synucleinopathies, aggregates from a low molecular weight form (LMW-αS) to a high molecular weight αS oligomer (HMW-αSo). Aggregated αS accumulates intracellularly, induces intrinsic apoptosis, is released extracellularly, and appears to propagate disease through prion-like spreading. Whether extracellular αS aggregates are cytotoxic, damage cell wall, or induce cell death is unclear. We investigated cytotoxicity and cell death caused by HMW-αSo or LMW-αS. Extracellular HMW-αSo was more cytotoxic than LMW-αS and was a crucial factor for inducing plasma membrane damage and cell death. HMW-αSo induced reactive oxygen species production and phospholipid peroxidation in the membrane, thereby impairing calcium homeostasis and disrupting plasma membrane integrity. HMW-αSo also induced extrinsic apoptosis and cell death by activating acidic sphingomyelinase. Thus, as extracellular HMW-αSo causes neuronal injury and death via cellular transmission and direct plasma membrane damage, we propose an additional disease progression pathway for α-synucleinopathies.

## Introduction

Parkinson’s disease (PD) is the second most common neurodegenerative disease. Its prevalence is >1% in individuals aged 65 years or older. Currently, approximately 6 million people worldwide are affected by PD, and it is estimated that the number of patients will double by 2050^1^. The pathology of PD is characterized by the accumulation of α-synuclein (αS) throughout the body. Brain αS aggregates cause motor symptoms, such as akinesia, muscle rigidity, and resting tremor, as well as various non-motor symptoms. Accumulation of αS is linked to diseases, such as dementia with Lewy bodies (DLB)^2^ and multiple system atrophy (MSA)^3^; such diseases are collectively termed “α-synucleinopathies.” αS normally exists in the pre-synaptic terminal as an unfolded monomer, whereas under pathological conditions, it aggregates and eventually forms intracellular inclusion bodies. An increase in the content of ganglioside 3 in the cell membrane and a high membrane curvature promote αS aggregation^4,5^. These αS aggregates are transmitted from cell to cell in a prion-like manner, affecting different tissues and neuroanatomically interconnected brain regions^6^. The distribution of αS oligomers differs from that of Lewy bodies in that αS oligomer burden is significantly greater in the neocortex, while that of Lewy bodies is greater in vulnerable subcortical regions, including the brainstem^7^. In addition, αS sequence variants generate different “strains,” the perpetuation of which would depend on the relative rates of aggregation and the relative stabilities of the aggregates formed by each strain^8^. Aggregated αS is internalized primarily by dynamin-mediated endocytosis^9^, and the internalized and accumulated αS aggregates are supposedly cytotoxic. In contrast, αS monomers are assumed to pass through the plasma membrane by direct translocation rather than by endocytosis^10^. There is much evidence of the transmission of extracellular αS, but the potential neurotoxic effect of αS from the extracellular side of the cell membrane has not been established. The role of soluble oligomers as early and intermediate aggregates of pathological proteins in the pathogenesis of neurodegenerative diseases, such as PD and Alzheimer’s disease (AD), has recently attracted attention^11,12^. Generally, the neurotoxic mechanisms associated with oligomers (αS, β amyloid, or tau) can be attributed to three fundamental processes: unspecific membrane interaction, interaction with specific entities, or membrane pore formation^13^.

The plasma membrane is regarded as the first barrier against the action of extracellular αS on the cell. Compared to fibrils and monomers, αS oligomers appear to have a higher affinity for and interact more avidly with the cell membrane^14^. In other amyloids, a two-step membrane disruption mechanism mediated by fibrils has been reported^15^. In addition, αS oligomers may interact directly with the neuronal membrane through specific receptors, pore formation, or unspecific interaction with the lipid bilayer structure^16^. Fluorescence resonance energy transfer analysis of oligomers by size (types A and B) shows that larger type B oligomers with rich β-sheet structures damage the plasma membrane^14,17^. Furthermore, type B oligomers are presumed to insert a β-sheet-rich core into the plasma membrane and disrupt its structure^14^. These αS oligomers induce oxidative stress more strongly than other molecules^17^. Thus, αS oligomers from the extracellular space, particularly the larger oligomers, could be involved in cellular injuries.

In α-synucleinopathies, dopaminergic neuron death is induced during αS accumulation, and apoptosis is considered one of its major mechanisms. In postmortem studies of patients with PD, DNA fragmentation and apoptotic chromatin changes in dopaminergic neurons have been observed, confirming evidence of apoptosis^18^. In MSA, apoptotic cell death has been identified exclusively in oligodendrocytes^19^. The high percentage of dopaminergic neurons with elevated caspase-3 activity in patients with PD suggests an association between caspase-dependent apoptosis and PD^20^. PD-inducing drugs, such as 1-methyl-4-phenyl-1,2,3,6-tetrahydropyridine and paraquat, are known to induce apoptosis by damaging mitochondria^21,22^. αS oligomers also interact with ATP synthase to open mitochondrial permeability transition pores^23^. Subsequently, cytochrome c is released from the mitochondria, mobilizing caspase-9 and inducing intrinsic apoptosis^24^. Although the apoptotic pathways induced by intracellular αS aggregates have been identified, whether extracellular αS aggregates can cause direct cytotoxicity via membrane damage remains unclear. Recently, it was reported that the soluble αS aggregates present in actual PD brains are smaller and more inflammatory than those present in control brains^25^. This suggests that non-fibrillar αS aggregates may be the critical species driving neuroinflammation and PD pathogenesis.

In this study, we investigated the mechanism of cytotoxicity and cell death caused by extracellular exposure of cells to high molecular weight αS oligomer (HMW-αSo) or low molecular weight αS (LMW-αS).

## Results

### Imaging of LMW-αS and HMW-αSo

**Fig. 1:**
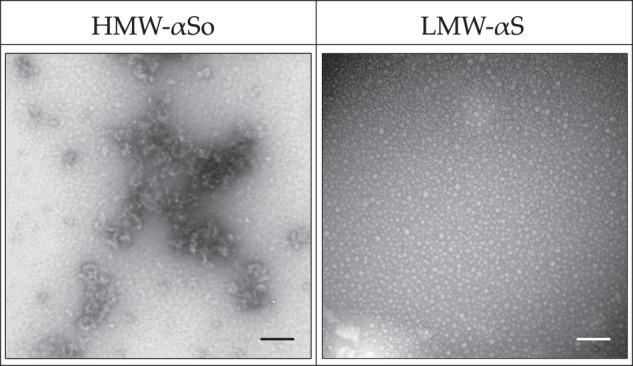
TEM images of HMW-αSo and LMW-αS. Scale bars, 100 nm.

Transmission electron microscopy was used to determine the morphologies of LMW-αS and HMW-αSo (Fig. 1). LMW-αS was a small globular structure with varying widths of 3‒25 nm. HMW-αSo appeared as aggregates, some of which were thread-like or bead-like, which matched the morphologies of protofibrils^26,27^.

**Fig. 2:**
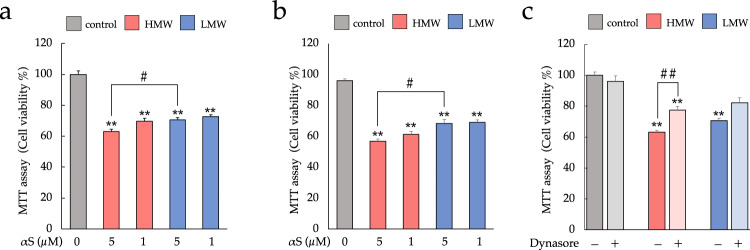
Effect of HMW-αSo and LMW-αS on cell viability in vitro. **a**–**c** MTT assay. **a** Cell viability of SH-SY5Y cells and (**b**) primary neurons exposed to αS for 24 h. **c** Cell viability of SH-SY5Y cells pretreated with dynasore, an αS endocytosis inhibitor, followed by exposure to αS. Values are expressed as mean + SEM. One-way ANOVA followed by Tukey’s post-hoc test (*n* = 10). **p < 0.01 vs. control cells.^#^*p* < 0.05, ^##^*p* < 0.01 vs. LMW-αS group.

To determine the neurotoxicity of HMW-αSo and LMW-αS, we evaluated the viability of SH-SY5Y cells and primary neurons using the MTT assay (Fig. 2a–c, Supplementary Fig. 1a–b). As shown in Fig. 2a, exposure to each αS assembly for 24 h resulted in a concentration-dependent decrease in viability of SH-SY5Y cells compared to that of controls, with 5 μM HMW-αSo reducing cell viability to a greater extent than 5 μM LMW-αS (Tukey, *p* = 0.03259). A reproducible, concentration-dependent decrease in cell viability was also observed in primary rat neurons, particularly when HMW-αSo was applied (Fig. 2b). Next, to distinguish the cytotoxicity of the internalized αS from that of the extracellular αS, cell viability experiments were performed in the presence of dynasore, an endocytosis inhibitor. Dynamin-dependent endocytosis is the main pathway for internalization of extracellular αS^9^, and dynasore is a dynamin inhibitor that suppresses αS internalization^28^. In Fig. 1c, the viability of SH-SY5Y cells was assessed in the presence or absence of dynasore pretreatment. Dynasore decreased but did not eliminate the toxicity of HMW-αSo. This suggests that cell damage caused by extracellular HMW-αSo may be mediated through both direct action at the cell membrane and internalization.

**Fig. 3:**
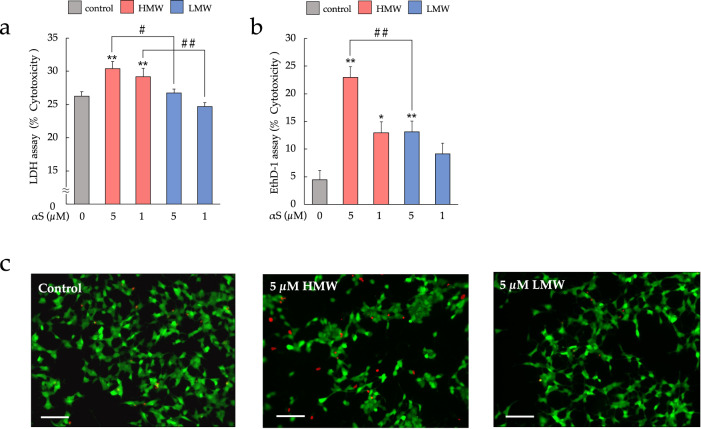
HMW-αSo induces cytotoxicity in SH-SY5Y cells. **a** LDH assay. Values are expressed as means + SEM. One-way ANOVA followed by Tukey’s post-hoc test (*n* = 10). **b** EthD-1 assay. Values are expressed as mean + SEM. One-way ANOVA followed by Tukey’s post-hoc test (*n* = 10). **c** Observations of control cells and each αS-exposed SH-SY5Y cell stained with calcein-AM (green) and EthD-1 (red). Scale bars, 100 µm. *p < 0.05, **p < 0.01 vs. control cells. ^#^*p* < 0.05, ^##^*p* < 0.01 vs. LMW-αS group.

To determine if frank cell lysis occurred with αS exposure, we measured lactate dehydrogenase (LDH) release (Fig. 3a). HMW-αSo-treated cells released significantly more LDH than did the control and LMW-αS-treated cells. The increase in membrane damage upon αS treatment correlated with the cell viability results of the MTT assay. We also used ethidium homodimer 1 (EthD-1, red fluorescence), a membrane-impermeable dye, which only enters cells with damaged membranes and binds to nucleic acids, to detect dead cells. In Fig. 3b, exposure to either type of αS significantly increased the percentage of EthD-1-positive SH-SY5Y cells compared to their fraction in control cells, especially upon treatment with 5 µM HMW-αSo which was significantly more toxic than LMW-αS at the same concentration (Tukey, *p* = 0.0048). We evaluated the individual morphology of αS-treated and untreated SH-SY5Y cells by co-staining with EthD-1 and calcein-AM, a live-cell stain (green fluorescence) (Fig. 3c). The cells exposed to HMW-αSo showed red fluorescent nuclei compared to the appearance of control and LMW-αS-treated cells, suggesting an increase in the number of dead cells. Taken together, HMW-αSo exhibits a higher cellular toxicity than LMW-αS.

### HMW-αSo induces oxidative stress and peroxidation of cell membranes

**Fig. 4:**
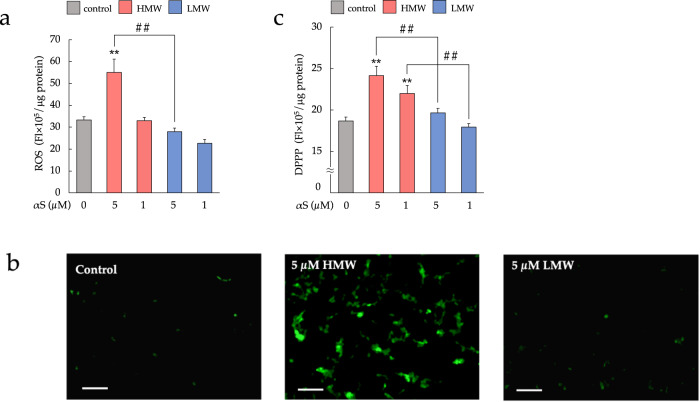
HMW-αSo induces oxidative stress. **a** ROS generation. Values are expressed as mean + SEM. One-way ANOVA followed by Tukey’s post-hoc test (*n* = 10). **b** Observation of SH-SY5Y cells by fluorescence of CM-H2DCFDA. Scale bars, 100 µm. **c** Diphenyl-1-pyrenylphosphine assay. Values are expressed as mean + SEM. One-way ANOVA followed by Tukey’s post-hoc test (*n* = 11). **p < 0.01 vs. control cells. ^##^*p* < 0.01 vs. LMW-αS group.

Oxidative stress is linked to mitochondrial depolarization, endoplasmic reticulum stress, and αS accumulation, thereby promoting PD progression^28^. It has also been suggested that αS oligomers, more so than monomers, are involved in reactive oxygen species (ROS) production^17^. To evaluate oxidative stress induced by extracellular αS, SH-SY5Y cells were treated with αS assemblies, and ROS generation was assessed. As shown in Fig. 4a, b, ROS production in SH-SY5Y cells was significantly increased after exposure to 5 μM HMW-αSo compared to that in control or LMW-αS-treated cells. Figure 4c presents results of monitoring peroxidation of plasma membrane phospholipids. Extracellular HMW-αSo induced significant phospholipid peroxidation compared to LMW-αS and controls. These data are in agreement with the results of ROS generation and further strengthen the postulation that extracellular HMW-αSo is involved in ROS generation and induce membrane damage through peroxidation of plasma membrane phospholipids.

### HMW-αSo reduces plasma membrane fluidity more than LMW-αS, depolarizes the membrane, and increases intracellular Ca^2+^ ([Ca^2+^]i)

**Fig. 5:**
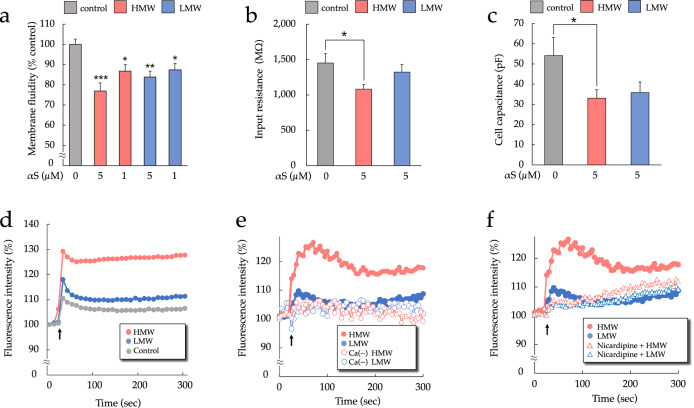
HMW-αSo disrupts cell membrane integrity. **a** Membrane fluidity. Each value is expressed relative to the control value (set to 100%). Values are expressed as mean + SEM. One-way ANOVA followed by Tukey’s post-hoc test (*n* = 20). **b**, **c** Electrophysiological changes measured using whole-cell patch-clamp recording. **b** Average input resistance and (**c**) whole-cell capacitance. Values are expressed as mean + SEM. One-way ANOVA followed by Tukey’s post-hoc test (*n* = 15). **d** Changes in membrane potential in SH-SY5Y cells exposed to 5 µM HMW-αSo or LMW-αS. Arrow indicates the addition of αS or control to cells. **e** Changes in [Ca^2+^]i were measured fluorometrically in untreated SH-SY5Y cells and in cells exposed to 5 µM HMW-αSo or LMW-αS. The arrow indicates the addition of αS to cells. Changes in [Ca^2+^]i during exposure to each αS were also measured in Ca^2+^-free buffer. **f** Changes in [Ca^2+^]i measured in SH-SY5Y cells pretreated with 10 µm nicardipine or untreated, followed by exposure to 5 µM HMW-αSo or LMW-αS. The arrow indicates the addition of αS to cells. *p < 0.05, **p < 0.01, ***p < 0.001 vs. control cells.

αS aggregates directly bound to membrane lipids appear to damage the phospholipid bilayer structure. αS oligomers have a high affinity for cell membranes and have been reported to have membrane-disrupting potential^14^. We examined changes in plasma membrane fluidity, [Ca^2+^]i, and membrane potential in SH-SY5Y cells. We also used whole-cell patch-clamp recording to monitor the effects of αS exposure on cellular resistance and capacitance. As shown in Fig. 5a, exposure to either αS significantly reduced plasma membrane fluidity, an effect that was largest with 5 µM HMW-αSo.

Electrophysiological monitoring of αS-exposed cells showed that HMW-αSo exposure reduced input resistance and cell capacitance (Fig. 5b, c). Next, membrane potential changes were measured using the membrane potential-sensitive dye DiBAC_4_(3) (Fig. 5d). Immediately after exposure to HMW-αSo, the DiBAC_4_(3) fluorescence intensity increased substantially. LMW-αSo also caused an increase, but of a lower magnitude. After a relatively rapid, modest decrease from the peak fluorescence intensity, all intensities remained stable during the experiment. These data indicated that HMW-αSo electrically destabilized cell membranes.

Since a substantial increase in [Ca^2+^]i causes dysfunction of cell organelles and apoptosis^29^, [Ca^2+^]i was analyzed after the addition of αS in Ca^2+^-free or Ca^2+^-containing buffer (Fig. 5e). Results were consistent with the fluorescence data. For both αS, [Ca^2+^]i increased immediately after the addition, with HMW-αSo showing the largest increase. However, in the Ca^2+^-free buffer, [Ca^2+^]i did not increase. [Ca^2+^]i changes under HMW-αSo or LMW-αS exposure were also measured in the presence of nicardipine, the L-type voltage-dependent Ca^2+^ channel blocker. Pretreatment with nicardipine (10 μM) suppressed, although not completely, the [Ca^2+^]i increase by HMW-αSo exposure (Fig. 5f).

### HMW-αSo induces apoptosis by damaging the cell membrane

**Fig. 6:**
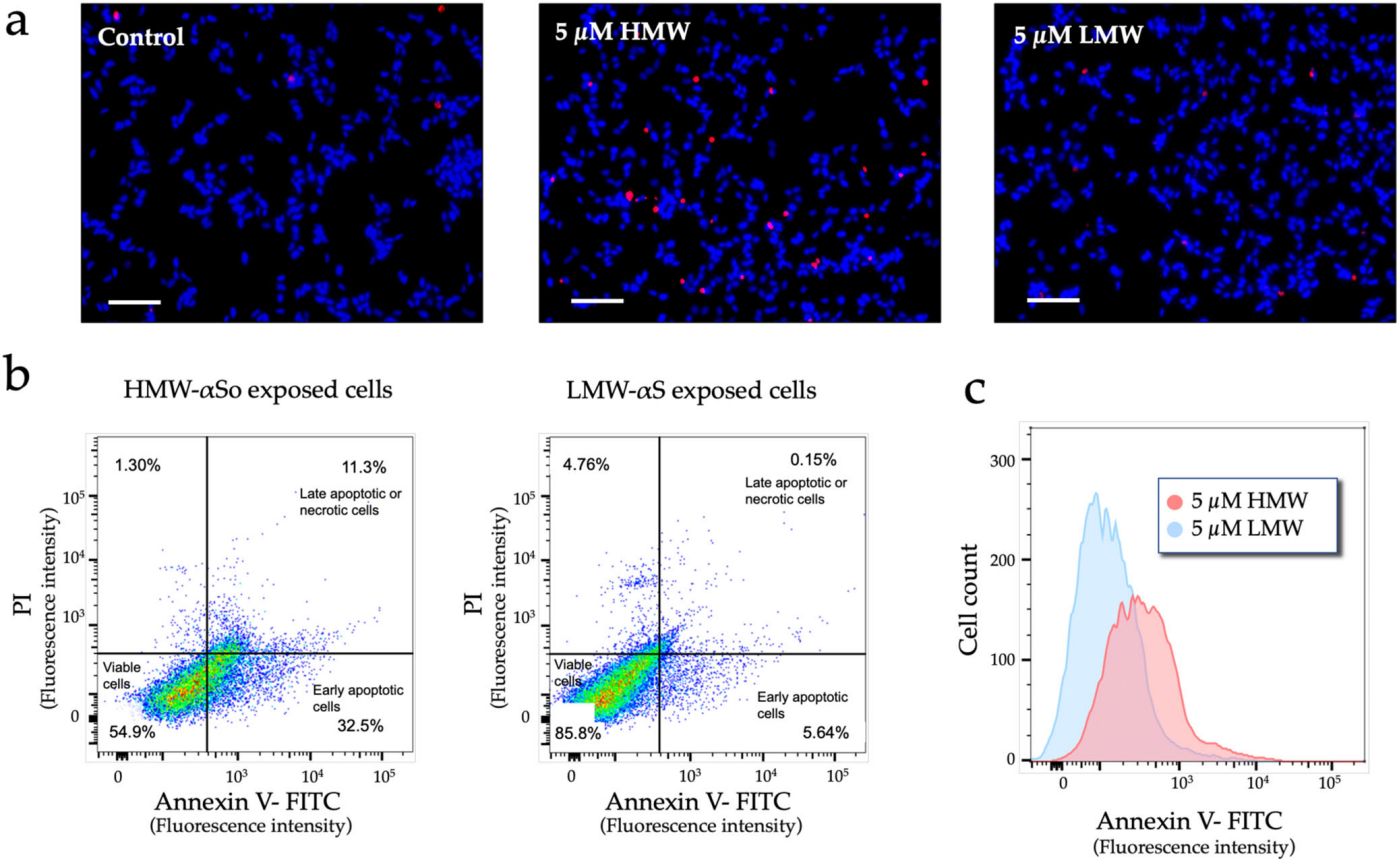
HMW-αSo induces apoptosis in SH-SY5Y cells. **a** Cells (living and dead) were identified by staining of their nuclei with Hoechst 33342. Apoptotic cells are indicated by Annexin V-FITC staining (pink). Scale bars, 100 µm. **b** Evaluation by flow cytometry in 5 µM HMW-αSo- or LMW-αS-exposed SH-SY5Y cells stained with Annexin V and propidium iodide (PI). The quadrants show viable cells (lower left), apoptotic cells (lower right), and late apoptotic or necrotic cells (upper right). Each percentage is expressed relative to the total number of cells targeted (set to 100%). **c** Histogram of the number of Annexin V stain-positive cells exposed to HMW-αSo or LMW-αS.

αS, especially in the oligomer form, induces apoptosis, which is considered a major cause of cell death in PD^30^. However, most studies have focused on organelle injury caused by internalized αS. Therefore, in this study, we examined whether apoptosis could also be induced by exposure to extracellular αS using fluorescein-labeled Annexin V (Annexin V-FITC), which binds to phosphatidylserine translocated from the inner to the outer leaflet (extracellular side) of the plasma membrane during apoptosis^31^. An increase in early apoptotic SH-SY5Y cells was observed after HMW-αSo exposure (Fig. 6a). To determine the relative numbers of early apoptotic, late apoptotic or necrotic, or viable cells, we double-labeled SH-SY5Y cells with Annexin V-FITC and propidium iodide (PI; to detect dead cells) and sorted these cells using flow cytometry (Fig. 6b). Cells exposed to HMW-αSo had a higher percentage of early apoptotic cells (FITC^+^/PI^−^) and a lower percentage of viable cells (FITC^−^/PI^−^) than did LMW-αS-treated cells. The proportion of late apoptotic or necrotic cells (FITC^+^/PI^+^) was also higher in HMW-αSo-exposed cells, consistent with the results of the EthD-1 assay. In addition, HMW-αSo-exposed cells, on average, fluoresced with higher intensities than LMW-αS-exposed cells (Fig. 6c).

**Fig. 7:**
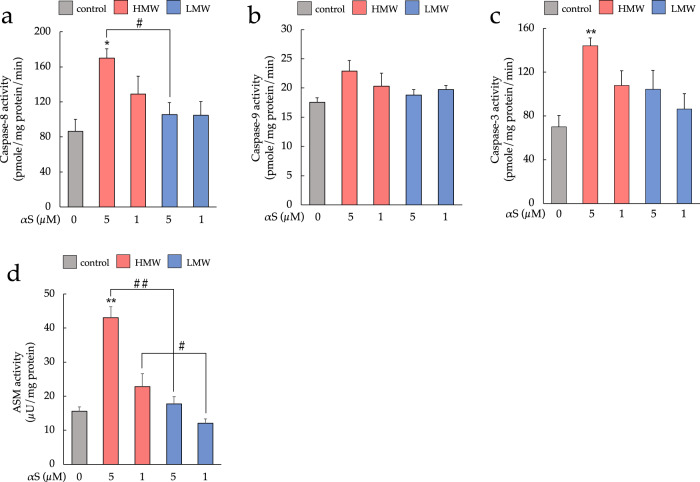
HMW-αSo induces cell death via damage to cell membranes. Activities of caspase-8 (**a**), 9 (**b**), and 3 (**c**) in SH-SY5Y cells exposed to HMW-αSo or LMW-αS. **d** Activation levels of acidic sphingomyelinase (ASM) induced by 5 µM HMW-αSo or LMW-αS. Values are expressed as mean + SEM. One-way ANOVA followed by Tukey’s post-hoc test (*n* = 10). *p < 0.05, **p < 0.01 vs. control cells. ^#^*p* < 0.05, ^##^*p* < 0.01 vs. LMW-αS group.

We then determined the activities of caspases-8, 9, and 3 to assess the caspase-dependent apoptosis pathway (Fig. 7a–c). In SH-SY5Y cells exposed to 5 µM HMW-αSo, the activity of caspase-8, an extrinsic apoptosis initiator, increased in a dose-dependent manner. Caspase-8 activity was significantly higher in HMW-αSo-treated cells than in LMW-αS-treated and control cells, while there was no significant difference between LMW-αS-treated and control cells. A trend of increasing activity was observed for caspase-9, an intrinsic apoptosis initiator, but the increase in activity was not statistically significant. Relative to that in controls, the activity of caspase-3, an executioner caspase, significantly increased in 5 µM HMW-αSo-treated cells. These results indicated that exposure to extracellular HMW-αSo activates the death receptor pathway on the plasma membrane and induces apoptosis. Support for this hypothesis was obtained from assays of sphingomyelinase activity, which revealed degradation of sphingomyelin (SM), a component of cell membranes (Fig. 7d). Acidic sphingomyelinase (ASM) mainly degrades SM and produces ceramide, an apoptosis-inducing factor. The relative activities of this enzyme were almost identical to those of caspase-8 (Fig. 7a), i.e., a dose-dependent increase in activity was observed in the presence of HMW-αSo. In summary, HMW-αSo not only activates the caspase-dependent apoptosis pathway by acting on death receptors on the plasma membrane (extrinsic apoptosis) but also induces ceramide production by increasing ASM activity and inducing caspase-independent apoptosis or necrosis.

## Discussion

**Fig. 8:**
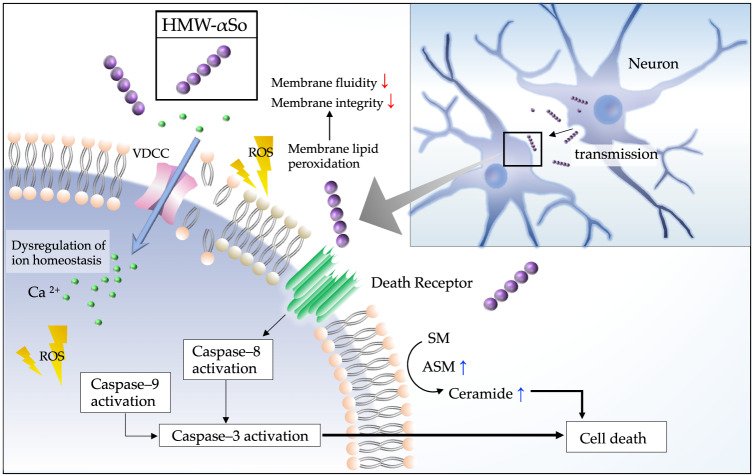
HMW-αSo induce oxidative stress, thereby reducing the plasma membrane fluidity and disrupting intracellular Ca^2+^ homeostasis. During membrane disruption, death receptors are stimulated, leading to extrinsic apoptosis. HMW-αSo increase ASM activity, producing ceramide and promoting cell death. HMW-αSo high molecular weight α-synuclein oligomer, VDCC voltage-dependent Ca^2+^ channel, ROS reactive oxygen species, SM sphingomyelin, ASM acidic sphingomyelinase, ↑; increase, ↓; decrease.

In this study, extracellular exposure of SH-SY5Y cells to HMW-αSo resulted in toxicity. The toxic effect resulted from plasma membrane damage and apoptosis mediated through cell death receptors and degradation of SM (Fig. 8). Our results are consistent with those of previous studies. In a postmortem study of patients with DLB, the levels of αS oligomers in the brain lysate were significantly higher than those in patients with AD^32^. In addition, αS oligomer levels in the cerebrospinal fluid are elevated in patients with PD^33,34^. Recently, detection of extracellular αS oligomers has been attempted in clinical practice. A combination of seed amplification assay and ELISA revealed that αS oligomer levels in the cerebrospinal fluid of patients with PD and DLB correlated with the Hoehn and Yahr or Unified Parkinson Disease Rating Scale motor scores^35^.

The mechanisms of αS oligomer-induced cytotoxicity have been found to involve mitochondrial dysfunction, endoplasmic reticulum stress, proteolytic system dysfunction, synaptic impairment, and neuroinflammation^11,27,36^. The first cell contact of αS oligomers is with the plasma membrane, where αS oligomers have been reported to have a higher membrane disruption ability than monomers or fibrils^14^. Both αS monomers and oligomers have an affinity for acidic, negatively charged lipids, but unlike monomers, αS oligomers are toxic.

Larger αS oligomers cause membrane disruption by inserting a β-sheet-rich core inside the lipid bilayer and inducing oxidative stress^14,17^. In addition, previous studies compared different αS oligomer species in terms of toxicity, calcium influx, and seeding and found that a higher toxicity was associated with smaller oligomers that were active inside the cells and capable of inducing pore formation^37,38^. It is hypothesized that oxidative stress is an important factor in PD progression^39^. Peroxidized cell membrane states have been shown to increase physiological and functional sensitivity to β-sheet-rich αS oligomers^40^. Furthermore, αS oligomers can induce a decrease in antioxidant substances, such as glutathione^41^.

The results of our experiments on ROS generation, peroxidation of the cell membrane, membrane fluidity, and membrane electrical properties are consistent with this notion. Changes in membrane properties because of oxidative stress may impair the function of intrinsic membrane proteins, such as channels, and thus alter ion homeostasis. Dysregulation of [Ca^2+^]i homeostasis is closely related to the pathogenesis of PD and AD^42^. Here, consistent with previous reports^43^, we demonstrated that HMW-αSo exposure increases cytosolic intracellular Ca^2+^ concentration through its effects on L-type voltage-dependent Ca^2+^ channels. However, we also evaluated the viability of SH-SY5Y cells with and without nicardipine pretreatment and found that nicardipine tended to reduce, but did not completely eliminate, the cytotoxicity caused by HMW-αSo (Supplementary Fig. 2a, b). Perhaps αS oligomer-induced cell injury is not limited to pathways initiated by disruption of Ca^2+^ homeostasis but involves multiple cell injury mechanisms, including membrane structural changes and cell death resulting from membrane damage.

HMW-αSo significantly increased caspase-8 and caspase-3 activities, but not caspase-9 activity, indicating that extracellular HMW-αSo exposure induced extrinsic apoptosis. However, under experimental conditions, there was no evidence of intrinsic apoptosis. A previous study showed that αS induced cell death via mitochondrial injury^44^. αS fibrils can induce intrinsic and extrinsic apoptosis during seeding^45^. Here, we focused on extracellular HMW-αSo species, which may explain why we did not observe intrinsic apoptosis, and the experimental conditions also differed between our study and the previous studies. It is unclear how death receptors that trigger extrinsic apoptosis were stimulated in this experiment. However, transmembrane death receptors are activated by ROS^46^, and it is possible that changes in membrane properties resulting from oxidative stress induced by HMW-αSo could have stimulated these receptors.

We also found that HMW-αSo increased ASM activity in SH-SY5Y cells and potentially stimulated the production of ceramide, a major factor in cell death. When activated by stimuli, such as oxidative stress, ASM migrates from lysosomes to the outer leaflet of the plasma membrane^47^, degrading SM in the plasma membrane and releasing ceramide, subsequently inducing cell death^48^. In addition, it is widely known that elevated glucocerebrosidase activity, which is involved in the hydrolysis of glucosylceramide into glucose and ceramide, is a risk factor for PD development^49^. There is evidence of increased ceramide levels in the autopsied brains of patients with PD compared to those in healthy controls^50^, and inhibition of ceramide synthesis in vitro reduced αS-induced cytotoxicity^51^. Ceramide is involved in various cellular damage mechanisms, including impairment of energy metabolism due to mitochondrial dysfunction, cellular senescence, and induction of autophagy^52^. Furthermore, the inhibition of neutral sphingomyelinase prevents dopaminergic neuron degeneration by preventing cell-to-cell transmission and exosome release of αS oligomers^53^. However, ASM deficiency causes Niemann-Pick disease owing to the accumulation of SM and is hypothesized to be a risk factor for PD development^54^. Although the alteration of ASM activity by HMW-αSo observed in this study will require confirmation and more detailed exploration, it is evident that changes in sphingoglycolipids have a significant impact on PD pathophysiology.

In conclusion, our study revealed that HMW-αSo directly damages plasma membrane from outside the cell, thereby causing neuronal death. This suggests that in addition to cell toxicity caused by intracellularly accumulated HMW-αSo, extracellular HMW-αSo can be toxic because of the damage it causes to the plasma membrane. The involvement of extracellular HMW-αSo in the pathophysiology of α-synucleinopathy suggests that these oligomers should be targeted in the development of disease-modifying therapies for PD.

## Methods

### Drugs and reagents

Dulbecco’s modified Eagle’s medium (DMEM) Ham’s F-12 and all-trans retinoic acid were purchased from FUJIFILM Wako Pure Chemical Corporation (Osaka, Japan). DMEM/F-12 medium, Neurobasal-A medium, antibiotic-antimycotic, fetal bovine serum (FBS), heat-inactivated horse serum, B-27 supplement, and Ara-c were obtained from Thermo Fisher Scientific (Waltham, MA, USA). Other chemicals used in this experiment were of the highest commercially available purity.

### Preparation of αS

Monomeric human αS was expressed in *Escherichia coli* BL21 (DE3) and purified as previously described^55^. The purity of the protein solution was confirmed to be >95% using MALDI-TOF mass spectroscopy (Bruker Daltonics, MA, USA). Purified human αS peptides were dissolved in DMEM Ham’s F-12 medium to prepare LMW-αS. To prepare HMW-αSo, the purified human αS peptides were dissolved in 10 mM phosphate buffer (pH 7.4) and adjusted to a final concentration of 840 μM. Then, several glass beads (ø 0.3–0.5 mm) were added as an aggregation seed and shaken constantly at 37 °C for 7 days. After incubation, the solution was centrifuged, the precipitates (mainly fibrils) were removed, and the supernatant was fractionated on a Superdex 200 increase 10/300 GL column (Merck & Co., Inc., NJ, USA) at a flow rate of 0.5 mL/min with 10 mM phosphate buffer. The HMW-αSo peak eluted at 27–28 min (Supplementary Fig. 3) was collected and stored at −80 °C. Protein concentrations were measured in each preparation using a Nano Orange Protein Quantitation kit (Thermo Fisher Scientific) according to the manufacturer’s instructions. The aggregate concentration was referenced to the monomeric αS concentration, with a typical HMW-αSo concentration of ~15 μM.

### Transmission electron microscopy

Aliquots of 10 µL from the HMW-αSo and LMW-αS fractions were dotted onto a glow-discharged carbon-coated formvar grid (Okenshoji, Tokyo, Japan) and incubated for 20 min. Then, an equal volume of 2.5% (vol/vol) glutaraldehyde solution was added dropwise, and the solution was incubated for 5 min. Finally, the proteins were stained with 8 µL of 1% (v/v) uranyl acetate (FUJIFILM Wako Pure Chemical Corporation). After removing the residual solution and air-drying the grids, the samples were observed under a transmission electron microscope (JEM-1210; JEOL Ltd., Tokyo, Japan).

### SH-SY5Y cell culture

SH-SY5Y cells (human neuroblastoma, EC-94030304) were obtained from the European Collection of Authenticated Cell Cultures (London, UK). The cells were cultured in DMEM Ham’s F-12 medium containing 10% FBS and antibiotic-antimycotic and maintained at 37 °C in a humid atmosphere of 5% CO_2_ and 95% air. We used SH-SY5Y cells differentiated for 7 days with 10 µM all-trans retinoic acid, which confers a predominantly mature dopaminergic-like neurotransmitter phenotype^56^. Differentiated SH-SY5Y cells were exposed to 5 µM or 1 µM HMW-αSo or LMW-αS for 24 h under sterile conditions.

### Primary neuron culture

Primary dissociated cultures were prepared from 1- or 2-day-old rats (Wistar, Nippon Bio-Supp. Center, Tokyo, Japan) as described previously^57^. The dissociated cells were seeded in collagen-coated 96-well plates at a final density of 1 × 10^5^ cells/mL. The culture medium (DMEM/F-12) contained 5% FBS, 5% heat-inactivated horse serum, and antibiotic-antimycotic. Neurobasal-A medium containing B27 supplement, Ara-c, and antibiotic-antimycotic was used as the serum-free medium for this experiment. In the present study, cells were exposed to 5 µM or 1 µM HMW-αSo or LMW-αS for 24 h under sterile conditions. This study was approved by the Ethics Committees of Showa University School of Medicine. All procedures of the study were approved by the Committee of Animal Care and Welfare of Showa University and were performed according to the Committee’s guidelines.

### Cell viability assay

The viability of SH-SY5Y cells and primary neurons exposed to different concentrations (5 µM or 1 µM) of HMW- and LMW-αS was evaluated using the MTT assay. The MTT Cell Count kit (Nacalai Tesque, Inc., Kyoto, Japan) was used according to the manufacturer’s instructions. After exposure of cells to HMW-αSo or LMW-αS for 24 h, MTT assays were performed, and absorbance was measured at 540 nm using a Spectra Max i3 microplate reader (Molecular Devices Co., CA, USA). To distinguish between cytotoxic effects caused by internalized and extracellular αS, MTT assays were performed after pretreatment with the αS endocytosis inhibitor dynasore (Abcam, Cambridge, UK). SH-SY5Y cells pretreated with 80 µM dynasore for 1 h were exposed to 5 μM HMW-αSo or LMW-αS for 24 h. Cell viability was measured and compared to that of cells treated with dynasore.

### Calcein-AM and EthD-1 cell assay

Cytotoxicity was measured by co-staining the cells with calcein-AM and EthD-1. SH-SY5Y cells were seeded in 96-well collagen-coated plates at a density of 1.0 × 10^6^ cells/mL, incubated at 37 °C for 24 h, and exposed to HMW-αSo or LMW-αS for 24 h. After incubation, the treated cells were stained with 2 µM calcein-AM and 10 µM EthD-1 (Thermo Fisher Scientific). Green fluorescence intensity was measured at an excitation wavelength of 495 nm and an emission wavelength of 530 nm using a Spectra Max i3 instrument (Molecular Devices). Red fluorescence intensity was measured at an excitation wavelength of 495 nm and an emission wavelength of 645 nm. The morphology of the individual cells was observed using a fluorescence microscope (BZ-X800; Keyence, Osaka, Japan).

### LDH release assay

Cytotoxic effects of HMW- and LMW-αS were also inferred from the amount of LDH released from cells with damaged membranes using an LDH Cytotoxicity Assay kit (Nacalai Tesque, Inc.) according to the manufacturer’s instructions. After exposure of cells to HMW-αSo or LMW-αS for 24 h, the formazan product produced by LDH and released into the medium was measured at a wavelength of 490 nm using a Spectra Max i3 instrument (Molecular Devices).

### ROS detection

To detect the effect of αS on ROS production, we used a chloromethyl derivative of the ROS-sensitive dye CM-H2DCFDA (Thermo Fisher Scientific). SH-SY5Y cells that were exposed to HMW-αSo or LMW-αS for 24 h were incubated for 15 min with CM-H2DCFDA at a final concentration of 6.5 µM. After washing each well, the fluorescence intensity at an excitation wavelength of 488 nm and an emission wavelength of 525 nm was measured using a Spectra Max i3 instrument (Molecular Devices) to estimate the amount of generated ROS. Cells in a state of oxidative stress caused by treatment with HMW-αSo or LMW-αS were observed using a fluorescence microscope (BZ-X800).

### Assay of phospholipid peroxidation in cell membranes

To evaluate phospholipid peroxidation in cell membranes, SH-SY5Y cells exposed to HMW-αSo or LMW-αS for 24 h were stained with 5 µM diphenyl-1-pyrenylphosphine (DPPP; Thermo Fisher Scientific) in Dimethyl sulfoxide at 37 °C for 10 min. Phospholipid peroxidation was then measured based on the fluorescence intensity of DPPP oxide at an excitation wavelength of 351 nm and an emission wavelength of 380 nm using a Spectra Max i3 instrument (Molecular Devices).

### Cell membrane fluidity

The membrane fluidity of SH-SY5Y cells was measured using pyrendecanoic acid, a lipophilic pyrene probe, using a Membrane Fluidity kit (Abcam) according to the manufacturer’s instructions. SH-SY5Y cells were seeded into 96-well plates at a density of 1.0 × 10^6^ cells/mL and exposed to HMW-αSo or LMW-αS for 24 h. Then, the treated cells were stained with pyrendecanoic acid, and membrane fluidity was measured according to a previously reported method^58^. The ratio of monomer (emission at 400 nm) to excimer (emission at 470 nm) fluorescence was measured using a Spectra Max i3 instrument (Molecular Devices).

### Changes in lipid membrane potential induced by DiBAC_4_ (3)

Changes in the membrane potential were monitored using bis (1,3-dibutylbarbituric acid) trimethine oxonol sodium salt (DiBAC_4_ (3); Dojindo Molecular Technologies, Inc., Kumamoto, Japan). Experiments were performed on SH-SY5Y cells seeded in 96-well plates and incubated in the assay buffer containing DiBAC_4_ (3) as previously described^59^. Cells were exposed to 5 μM HMW-αSo or LMW-αS, and changes in the membrane potential were measured every 10 s for 5 min at an excitation wavelength of 490 nm and emission wavelength of 516 nm using Flex Station 3 (Molecular Devices). Fluorescence intensity immediately before αS application was considered as 100%.

### Whole-cell patch-clamp recording

SH-SY5Y cells were cultured to 50–70% confluency in poly-D-lysine-coated glass bottom dishes (MatTek, Ashland, MA, USA) for the experiments. The cells were then exposed to each αS (5 µM) for 30 min. Current clamp measurements were performed using a Multiclamp 700 B amplifier (Molecular Devices) according to previously described methods^58^.

### [Ca^2+^]i measurements

Intracellular Ca^2+^ ([Ca^2+^]i) levels in SH-SY5Y cells were measured using a FLIPR Calcium 5 Assay kit (Molecular Devices) according to the manufacturer’s instructions. SH-SY5Y cells were loaded with FLIPR reagent containing 20 mM HEPES and 1 × Hank’s Balanced Salt Solution (containing 1.26 mM CaCl_2_, pH 7.4) at 37 °C for 60 min. Cells were exposed to 5 μM HMW-αSo or LMW-αS in the presence or absence of extracellular calcium (0 mM Ca^2+^ HBSS supplemented with HEPES and 0.3 mM EGTA). In addition, the cells were pretreated with 10 μM nicardipine (Thermo Fisher Scientific) for 5 min, followed by treatment with 5 μM HMW-αSo or LMW-αS. Fluorescence intensity immediately before administration was set at 100%.

### Annexin V staining for apoptosis detection

The Annexin V-Cy3 Apoptosis Detection kit (Merck & Co., Inc.) was used to determine the number of apoptotic cells after treatment with HMW-αSo or LMW-αS. Differentiated SH-SY5Y cells exposed to 5 µM HMW-αSo or LMW-αS for 24 h were stained with Hoechst 33342 and Annexin V-Cy3, and then observed under a fluorescence microscope (BZ-X800). In addition, the cells were co-stained with Annexin V-FITC and PI (Thermo Fisher Scientific) and identified as early or late apoptotic and necrotic cells using a BD FACSLyric flow cytometer (Becton Dickinson, NJ, USA).

### Measurement of caspases activities

To determine the effect of extracellular αS on the caspase-dependent apoptotic pathway, activities of caspases 8, 9, and 3 were evaluated according to the cleavage of their respective substrates. Lysates of differentiated SH-SY5Y cells exposed to HMW-αSo or LMW-αS for 24 h were prepared, and a reaction buffer containing 10 mM dithiothreitol (Abcam) was added. IETD-AFC, LEHD-AFC, and DEVD-AFC (Abcam), fluorogenic substrates for caspases 8, 9, and 3, respectively, were added. AFC released by the enzyme reaction was measured at an excitation wavelength of 400 nm and an emission wavelength of 505 nm using a Spectra Max i3 instrument (Molecular Devices) at 37 °C, every 30 min for 3 h.

### Measurement of ASM activity

An Amplite^TM^ Fluorimetric ASM Assay kit (AAT Bioquest, Inc., CA, USA) was used to measure the level of ASM produced by the cells after exposure to HMW-αSo or LMW-αS. SH-SY5Y cells cultured in 96-well plates were exposed to HMW-αSo or LMW-αS for 24 h. The cells were then lysed and reacted with a fluorescent probe to indirectly measure ASM activity by measuring the amount of phosphocholine.

### Statistical analysis

Statistical analysis was performed using JMP Pro 17 software for Windows (SAS Institute Inc., NC, USA). Each measurement was performed in triplicate. The results of all experiments are expressed as the mean ± standard error of the mean. The values of various parameters after treatment with HMW-αSo or LMW-αS were compared to those in untreated SH-SY5Y cells using one-way analysis of variance (ANOVA) followed by the Tukey’s post-hoc test. Effects were considered statistically significant at *p* < 0.05.

### Reporting summary

Further information on research design is available in the Nature Research Reporting Summary linked to this article.

### Supplementary information

Supplementary Figure 1

Reporting Summary

## Data Availability

All data used for this study is available from the corresponding author on request.
