# Supplementary material for: Extracellular high molecular weight α-synuclein oligomers induce cell death by disrupting the plasma membrane
Source: NPJ Parkinsons Dis. 2023 Sep 28;9:139. doi: 10.1038/s41531-023-00583-0 (PMC10539356; doi:10.1038/s41531-023-00583-0)
Supplement: Supplementary file 1 — Supplementary Figure 1 [file 41531_2023_583_MOESM1_ESM.pdf]

Supplementary Figure

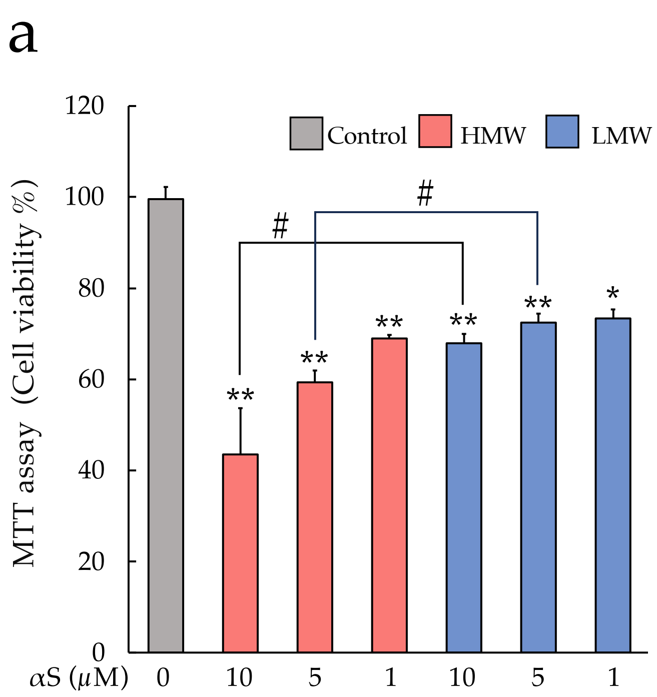

Supplementary Figure 1. Effect of HMW-αSo and LMW-αS on cell viability in vitro. (a,b) MTT assay. (a) Cell viability of SH-SY5Y cells and (b) primary neurons exposed to αS for 24 h. Values are expressed as mean + SEM. One-way ANOVA followed by Tukey's post-hoc test (n = 10). * $p < 0.01$, **$p < 0.001$ vs. control cells. # $p < 0.05$ vs. LMW-αS group.

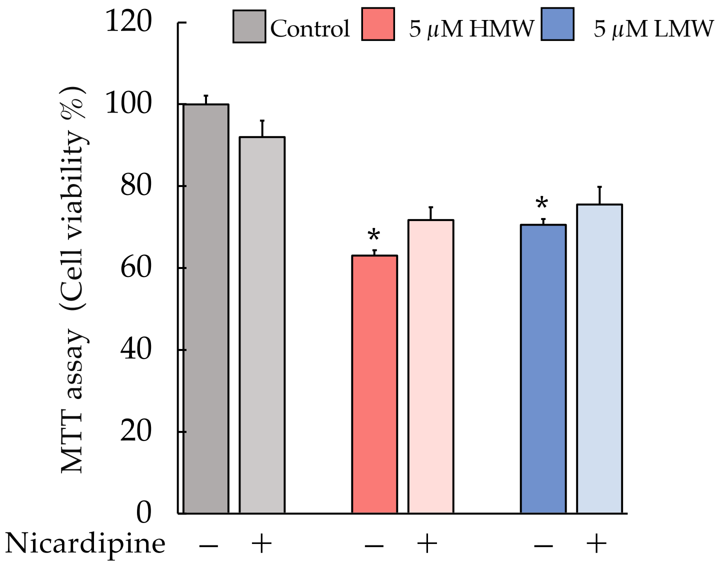

Supplementary Figure 2. Cell viability of SH-SY5Y cells pretreated with 10 μM nicardipine, an L-type calcium channel blocker, followed by exposure to αS. Values are expressed as means + SEM. One-way ANOVA followed by Tukey's post hoc test (n=10). *$p < 0.01$ vs. control cells.

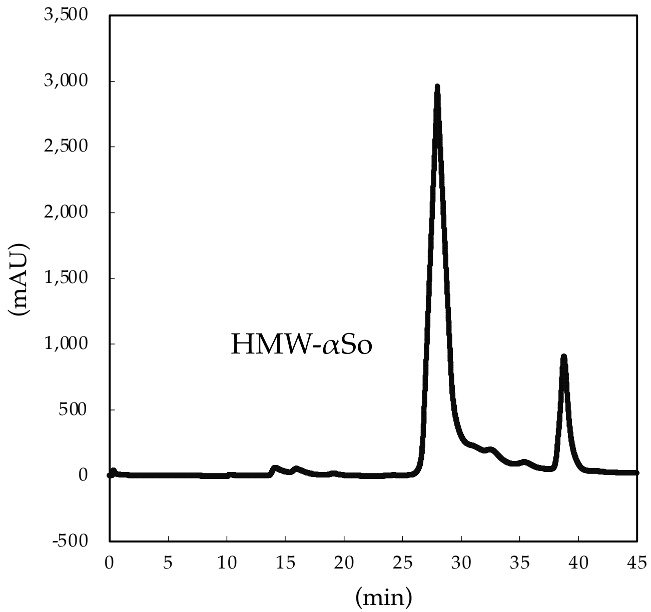

Supplementary Figure 3. HMW-αSo preparation. SEC of αS. The synthesized αS was applied on a gel filtration column. The HMW-αSo peak was eluted at 27–28 min.
