# Supplementary material for: Extracellular high molecular weight α-synuclein oligomers induce cell death by disrupting the plasma membrane
Source: NPJ Parkinsons Dis. 2023 Sep 28;9:139. doi: 10.1038/s41531-023-00583-0 (PMC10539356; doi:10.1038/s41531-023-00583-0)
Supplement: Supplementary file 2 — Reporting Summary [file 41531_2023_583_MOESM2_ESM.pdf]

nature portfolio

Corresponding author(s): Kenjiro Ono

Last updated by author(s): 2023-9-15

Reporting Summary

Nature Portfolio wishes to improve the reproducibility of the work that we publish. This form provides structure for consistency and transparency in reporting. For further information on Nature Portfolio policies, see our Editorial Policies and the Editorial Policy Checklist.

Statistics

For all statistical analyses, confirm that the following items are present in the figure legend, table legend, main text, or Methods section.

n/a	Confirmed	
☐	☒	The exact sample size (*n*) for each experimental group/condition, given as a discrete number and unit of measurement
☐	☒	A statement on whether measurements were taken from distinct samples or whether the same sample was measured repeatedly
☐	☒	The statistical test(s) used AND whether they are one- or two-sided<br>*Only common tests should be described solely by name; describe more complex techniques in the Methods section.*
☐	☒	A description of all covariates tested
☐	☒	A description of any assumptions or corrections, such as tests of normality and adjustment for multiple comparisons
☐	☒	A full description of the statistical parameters including central tendency (e.g. means) or other basic estimates (e.g. regression coefficient) AND variation (e.g. standard deviation) or associated estimates of uncertainty (e.g. confidence intervals)
☐	☒	For null hypothesis testing, the test statistic (e.g. F, t, r) with confidence intervals, effect sizes, degrees of freedom and P value noted<br>*Give P values as exact values whenever suitable.*
☒	☐	For Bayesian analysis, information on the choice of priors and Markov chain Monte Carlo settings
☒	☐	For hierarchical and complex designs, identification of the appropriate level for tests and full reporting of outcomes
☒	☐	Estimates of effect sizes (e.g. Cohen's *d*, Pearson's *r*), indicating how they were calculated

Our web collection on statistics for biologists contains articles on many of the points above.

Software and code

Policy information about availability of computer code

Data collection	The protein solutions' purity was confirmed using MALDI-TOF mass spectrometry (Bruker Daltonics, MA, USA). Absorbance and fluorescence measurements were conducted with a SpectraMax i3 instrument (Molecular Devices Co., CA, USA). Changes in lipid membrane potential induced by DiBAC4 (3) and intracellular Ca2+ levels in SH-SY5Y cells were recorded over time using a FlexStation 3 instrument (Molecular Devices). Transmission electron microscopy (JEM-1210; JEOL Ltd., Tokyo, Japan) was employed to observe the generated α-synuclein. Fluorescence microscopy (BZ-X800; Keyence, Osaka, Japan) was used for the observation of fluorescently stained cells. Membrane potentials were recorded in current-clamp configuration with the Multi- clamp 700B amplifier (Molecular Devices). The data were low- pass filtered at 10 kHz, sampled at 20 kHz (Digidata 1440; Molecular Devices). Flow cytometry was conducted using a BD FACSLyric flow cytometer (Becton Dickinson, NJ, USA).
Data analysis	Statistical analysis was performed using JMP Pro 17 software for Windows (SAS Institute Inc., NC, USA). The results of flow cytometry were analyzed using FlowJo™ v10.9 software (Becton Dickinson, NJ, USA). The results of whole-cell patch-clamp recording were analyzed using pCLAMP 10.7 (Molecular Devices), Origin 2016 (Origin-Lab Corporation, MA, USA).

For manuscripts utilizing custom algorithms or software that are central to the research but not yet described in published literature, software must be made available to editors and reviewers. We strongly encourage code deposition in a community repository (e.g. GitHub). See the Nature Portfolio guidelines for submitting code & software for further information.

Data

Policy information about availability of data

All manuscripts must include a data availability statement. This statement should provide the following information, where applicable:

- Accession codes, unique identifiers, or web links for publicly available datasets
- A description of any restrictions on data availability
- For clinical datasets or third party data, please ensure that the statement adheres to our policy

All data used for this study is available from the corresponding author on request.

Research involving human participants, their data, or biological material

Policy information about studies with human participants or human data. See also policy information about sex, gender (identity/presentation), and sexual orientation and race, ethnicity and racism.

Reporting on sex and gender	Not applicable.
Reporting on race, ethnicity, or other socially relevant groupings	Not applicable.
Population characteristics	Not applicable.
Recruitment	Not applicable.
Ethics oversight	Not applicable.

Note that full information on the approval of the study protocol must also be provided in the manuscript.

Field-specific reporting

Please select the one below that is the best fit for your research. If you are not sure, read the appropriate sections before making your selection.

☒ Life sciences ☐ Behavioural & social sciences ☐ Ecological, evolutionary & environmental sciences

For a reference copy of the document with all sections, see nature.com/documents/nr-reporting-summary-flat.pdf

Life sciences study design

All studies must disclose on these points even when the disclosure is negative.

Sample size	The cells were categorized into five groups: exposure to 5 µM HMW-αSo (high molecular weight α-synuclein oligomers), 1 µM HMW-αSo, 5 µM LMW-αS (low molecular weight α-synuclein), 1 µM LMW-αS, and control groups.
Data exclusions	All samples were analyzed, except for electron microscope images and fluorescent-stained images. For electron microscope images and fluorescence-stained images, a minimum of 20 fields of view were examined for each, and the most representative photographs were included.
Replication	Each measurement was conducted in triplicate.
Randomization	Cells were assigned randomly to experimental and control groups.
Blinding	The investigators were not blinded during data collection.

Reporting for specific materials, systems and methods

We require information from authors about some types of materials, experimental systems and methods used in many studies. Here, indicate whether each material, system or method listed is relevant to your study. If you are not sure if a list item applies to your research, read the appropriate section before selecting a response.

Materials & experimental systems

n/a	Involved in the study	
☒	☐	Antibodies
☐	☒	Eukaryotic cell lines
☒	☐	Palaeontology and archaeology
☐	☒	Animals and other organisms
☒	☐	Clinical data
☒	☐	Dual use research of concern
☒	☐	Plants

Methods

n/a	Involved in the study	
☒	☐	ChIP-seq
☐	☒	Flow cytometry
☒	☐	MRI-based neuroimaging

Eukaryotic cell lines

Policy information about cell lines and Sex and Gender in Research

Cell line source(s)	SH-SY5Y cells (human neuroblastoma, EC-94030304) were sourced from the European Collection of Authenticated Cell Cultures (London, UK).
Authentication	At the outset of the study, authentication was conducted through STR analysis.
Mycoplasma contamination	Mycoplasma was not detected through oligonucleotide-directed amplification testing.
Commonly misidentified lines (See ICLAC register)	No misidentified lines were used.

Animals and other research organisms

Policy information about studies involving animals; ARRIVE guidelines recommended for reporting animal research, and Sex and Gender in Research

Laboratory animals	1- or 2-day-old rats (Wistar, Nippon Bio-Supp. Center, Tokyo, Japan)
Wild animals	Not applicable.
Reporting on sex	The sex of neonatal rats is unspecified.
Field-collected samples	Not applicable.
Ethics oversight	This study was approved by the Ethics Committees of Showa University School of Medicine (the chairperson: Masahiko Izumizaki MD, Ph.D., certificate number: 09048, approved on April 1st 2019). All procedures of the study were approved by the Committee of Animal Care and Welfare of Showa University and were performed according to the Committee's guidelines.

Note that full information on the approval of the study protocol must also be provided in the manuscript.

Flow Cytometry

Plots

Confirm that:

☒ The axis labels state the marker and fluorochrome used (e.g. CD4-FITC).

☒ The axis scales are clearly visible. Include numbers along axes only for bottom left plot of group (a 'group' is an analysis of identical markers).

☒ All plots are contour plots with outliers or pseudocolor plots.

☒ A numerical value for number of cells or percentage (with statistics) is provided.

Methodology

Sample preparation	Differentiated SH-SY5Y cells were plated in 6-well collagen-coated plates at a density of 5.0×10^5 cells/mL. They were then incubated at 37 °C for 24 hours and exposed to HMW-αSo or LMW-αS for an additional 24 hours. Following incubation, the treated cells were stained with Annexin V-FITC and PI (Thermo Fisher Scientific,MA, USA) following the manufacturer's instructions. Apoptosis induction was assessed using flow cytometry by measuring the fluorescence of Annexin V-FITC and PI.
Instrument	BD FACSLyric flow cytometer (Becton Dickinson, NJ, USA)
Software	FlowJo™ v10.9 (Becton Dickinson, NJ, USA)

Cell population abundance	Not applicable.
Gating strategy	Forward Scatter (FSC) and Side Scatter (SSC) light were employed for initial gating to distinguish individual cells within the sample. This step excluded negative populations, such as cellular debris and fragments. Annexin V-FITC and PI fluorescence intensity were then used to gate positive and negative cells, allowing for the identification of early apoptotic cells, late apoptotic and necrotic cells using flow cytometry.

☐ Tick this box to confirm that a figure exemplifying the gating strategy is provided in the Supplementary Information.
